# Systematic review and meta-analysis of women's awareness of obstetric fistula and its determinants in Ethiopia

**DOI:** 10.3389/fgwh.2023.1151083

**Published:** 2023-05-19

**Authors:** Tamirat Melis, Ayenew Mose

**Affiliations:** ^1^ Department of Public Health, College of Medicine and Health Science, Wolkite University, Wolkite, Ethiopia; ^2^ Department of Midwifery, College of Medicine and Health Science, Wolkite University, Wolkite, Ethiopia

**Keywords:** obstetric fistula, women's awareness, systematic review, meta-analysis, Ethiopia

## Abstract

**Background:**

Although obstetric fistula has been extensively eliminated in high-income countries due to comprehensive obstetric health care services, in developing countries including Ethiopia, many women and girls are still silently suffering from obstetric fistula due to early marriage, poor socioeconomic status, lack of access to skilled birth attendants, and limited awareness of obstetric fistula.

**Objective:**

To determine the magnitude of women's awareness of obstetric fistula and its contributing factors in Ethiopia.

**Methods:**

To perform this analysis, we strictly adhered to the Preferred Reporting Items for Systematic Review and Meta-Analysis (PRISMA) recommendations. To evaluate publication bias, we employed an Egger's test and an eye assessment of the funnel plot's symmetry. To look for signs of study heterogeneity, the Cochrane *Q*-test and I2 statistics were used. A Microsoft Excel spreadsheet was used to extract the data, and STATA version 14 was used to analyze it.

**Results:**

A total of six studies involving 3,024 women were included. The pooled prevalence of women's awareness of obstetric fistula in Ethiopia was 41.24% (95% CI; 32.94%−49.54%). Urban residence (AOR = 2.32, 95% CI: 1.40–3.85), giving birth at a health institution (AOR = 2.84, 95% CI: 1.92–4.21), having secondary or above educational status (AOR = 3.27, 95% CI: 2.15–4.97), receiving antenatal care follow-up (AOR = 2.73, 95% CI: 1.71–4.35), and participation in pregnant women's conferences (AOR = 4.64, 95% CI: 2.88–7.49) were factors associated with good awareness of obstetric fistula in women in Ethiopia.

**Conclusion:**

The pooled prevalence of women's awareness of obstetric fistula was low. Urban residence, giving birth at a health institution, having secondary and above educational status, having antenatal care follow-up, and participating in pregnant women's conferences were factors associated with women's awareness of obstetric fistula. Therefore, enhancing women's awareness of obstetric fistula and promoting institutional delivery and antenatal follow-up is recommended. Furthermore, policymakers and stakeholders should empower women and pay particular attention to the neglected but important public health problem that is obstetric fistula.

## Background

Obstetric fistula (OBF) is a debilitating maternal mortality that results from childbirth injury mainly due to prolonged and obstructed labor, typically in the absence of skilled birth attendants (1, 2). In the case of prolonged labor, there is prolonged pressure of the fetal head on the maternal blood vessels supplying the tissues of the vagina, bladder, urethra, and rectum. Following delayed labor, blood vessels are easily damaged and, ultimately, ischemia can occur, cutting off the oxygen supply, which, regrettably, leads to necrosis. Subsequently, a hole between the vagina and the bladder (vesicovaginal fistula) or rectum (rectovaginal fistula) might develop and is referred to as OBF (3, 4).

Worldwide, an estimated 2–3 million women have OBF, indicating that 50,000–100,000 women develop this condition every year. Exclusively, more than 2 million young women live with untreated OBF in Asia and sub-Saharan Africa (5). In Ethiopia, 9,000 women develop OBF every year, 100,000 women are living with untreated fistula, and only 1,200 women receive treatment per year (6, 7).

In developed countries, the obstetric fistula has been virtually eliminated due to access to quality essential and comprehensive obstetric health care services (8) However, in developing countries, many young girls still silently suffer from obstetric fistula due to early marriage, poor socioeconomic status, and lack of access to a skilled birth attendant (9, 10). The “fistula belt,” which stretches from the northern half of sub-Saharan Africa to the southern part of Asia, bears the majority of the burden of obstetric fistula (11, 12).

A systematic review of the qualitative and mixed studies conducted in sub-Saharan Africa shows that women who develop obstetric fistula often face divorce, economic incapability, depression, and stigmatization by the community, leading to women committing suicide (13, 14). Women with obstetric fistula often live as outcasts, subjected to constant incontinence, shame, social segregation, and rejection by their families (15, 16). Furthermore, fistula can result in long-term health issues, such as leg ulcers, kidney illness, and nerve damage, and in severe cases, it can cause paralysis (17).

Significant evidence is available concerning the prevalence of obstetric fistula in Ethiopia (18, 19). However, limited studies are available regarding women's awareness of obstetric fistula, particularly on the risk factors, its prevention, and healthcare-seeking behavior.

Several global movements such as the UNFPA Campaign to End Fistula and the International Day to End Obstetric Fistula have carried out extensive activities to eradicate OBF (20, 21). Additionally, the UN aims to end fistula by 2030 (22). Although obstetric fistula is almost entirely preventable, it is still a huge public health issue in Ethiopia. To this end, women's awareness of obstetric fistula is crucial for primary prevention and to enhance women's healthcare-seeking behavior with the aim of early treatment. Therefore, this review is needed given the dearth of evidence at the national level regarding women's awareness of OBF and its associated factors. The findings have the potential to assist stakeholders and policymakers in improving women's awareness and eliminating new cases of obstetric fistula.

## Methods

### Study design and setting

A systematic review and meta-analysis were done to determine the overall magnitude of women's awareness of obstetric fistula and its contributing factors in Ethiopia. This review follows the PRISMA (Preferred Reporting Items for Systematic Review and Meta-Analysis) guidelines (Supplementary S1 File) (23). Furthermore, we checked the PROSPERO database (http://www.library.ucsf.edu/) to identify whether there are any relevant published or ongoing studies in order to prevent any future duplication. The findings revealed that there were no ongoing or published articles on this area of the topic and, as a result, this review's protocol was registered in the PROSPERO database.

### Search strategies and sources of information

International databases such as PubMed, African Online Journal, Web of Sciences, and Google Scholar were searched to retrieve related articles. Search terms were formulated using PICO guidelines through the online databases . The Boolean operators “AND” and “OR” were used to build the Medical Subject Headings (MeSH) and key phrases. The following search strategies were used for advanced PubMed searches: “Awareness” OR “Knowledge” AND “women” OR “Reproductive age group” AND “Obstetric fistula” OR “Rectovaginal fistula” OR “Vesicovaginal fistula” AND “Associated Factors” OR “Determinants” OR “Predictors” AND “Ethiopia”. We also searched using the title itself, “The magnitude and factors associated with women’s awareness of obstetric fistula in Ethiopia”.

### P: patient/population

✓ Reproductive-age women residing in Ethiopia
✓ Both institution and community were the setting

### I: intervention “exposure”

✓ Risk factor or exposing or precipitating factors for women's awareness in obstetric fistula are considered as intervention or exposure

### C: comparator

✓ Those in the study population who are not exposed to precipitating factors for women's awareness of obstetric fistula are comparators

### O: outcome

✓ The primary outcome was the status of women's awareness of obstetric fistula (good or poor awareness). Women's awareness of obstetric fistula is defined as being conscious of or informed about obstetric fistula risk factors, methods of prevention, and treatment approaches. Therefore, all included studies assessed the study participants' level of awareness by categorizing them as having poor awareness if they scored less than the mean value and good awareness if they scored above the mean value. The secondary outcome was factors associated with women's awareness of obstetric fistula.

#### Inclusion and exclusion criteria

Inclusion criteria for studies in this systematic review and meta-analysis were as follows: firstly, studies needed to report the prevalence of women's awareness of obstetric fistula and its associated factors; secondly, both published and unpublished articles, including pre-print studies, conducted at any time and written in the English language only were considered. Moreover, regarding the study period, there was no restriction, and all cross-sectional research conducted in Ethiopia up until the final day of the research period (April 14, 2022) was included. Articles reported outside the result interest and articles without full abstracts or texts were the exclusion criteria. Additionally, after reviewing the abstract and full texts, citations without complete texts and/or abstracts, qualitative research, comments, anonymous reports, letters, editorials, and reviews were disregarded.

### Outcome measurements

The primary outcome was the level of women's awareness of obstetric fistula (good or poor awareness). Women's awareness of obstetric fistula is defined as being conscious of or informed about obstetric fistula risk factors, methods of prevention, and treatment approaches. Therefore, all included studies assessed the study participant's level of awareness by categorizing them as having poor awareness if they scored less than the mean value and good awareness if they scored above the mean value. The secondary outcome was factors associated with women's awareness of obstetric fistula.

### Data extraction

The Endnote version X8 program was used to export every study that was obtained from the four databases. The complete texts of articles identified as possibly appropriate for inclusion were extracted after all duplicate abstracts were removed and independently examined by (TM and AM). Then, two authors (TM and AM) used a standardized data extraction form that was modified from the Joanna Briggs Institute (JBI) data extraction format to independently collect all the crucial data (24). For the first outcome (women's awareness of obstetric fistula), the data extraction format included primary author, survey period, year of study, year of publication, regions, sample size, study design, study setting, prevalence with 95% CI, and quality of each study. Two authors (TM and AM) extracted data for the second outcome (associated factors of women's awareness of obstetric fistula) using a 2 × 2 table format. Finally, the log odds ratio for each factor was calculated using STATA Version 14.0 Software.

### Quality assessment

The systematic review and meta-analysis assessed the quality of each study using the modified Newcastle Ottawa Quality Assessment Scale (NOS) for cross-sectional studies (Supplementary S2 File) (25). Two authors (TM and AM) evaluated the validity of each study, i.e., methodological quality, sample selection, sample size, comparability and the outcome, and statistical analysis of the study. Disagreements between the authors were resolved by consensus. The study design of all included articles in this study was cross-sectional.

### Data processing and analysis

Statistical analysis was carried out using STATA version 14 after selected articles were entered into a Microsoft Excel spreadsheet format. A weighted inverse variance random effect model was used to estimate the overall magnitude of women's awareness of obstetric fistula in Ethiopia. Because there was heterogeneity between studies (*I*^2^ > 50%), we utilized the random effect model; however, in the case of homogenous research, we used the fixed effect model. *I*^2^ statistics and the Cochrane *Q*-test were computed to determine the degree of study heterogeneity. Accordingly, mild heterogeneity was defined as 0%–40%, moderate heterogeneity as 30%–60%, substantial heterogeneity as 50%–90%, and considerable heterogeneity as 75%–100% (26). An Egger's test and a funnel plot were used to evaluate publication bias (27, 28). There was no publication bias, as the *p*-value was more than 0.05. Based on the study region, subgroup analysis was performed. A forest plot format was used to present the pooled prevalence of women's awareness of obstetric fistula with 95% CI. We used STATA version 14.0 software to identify associated factors with women's awareness of obstetric fistula.

## Results

### Characteristics of included studies

**Figure 1:**
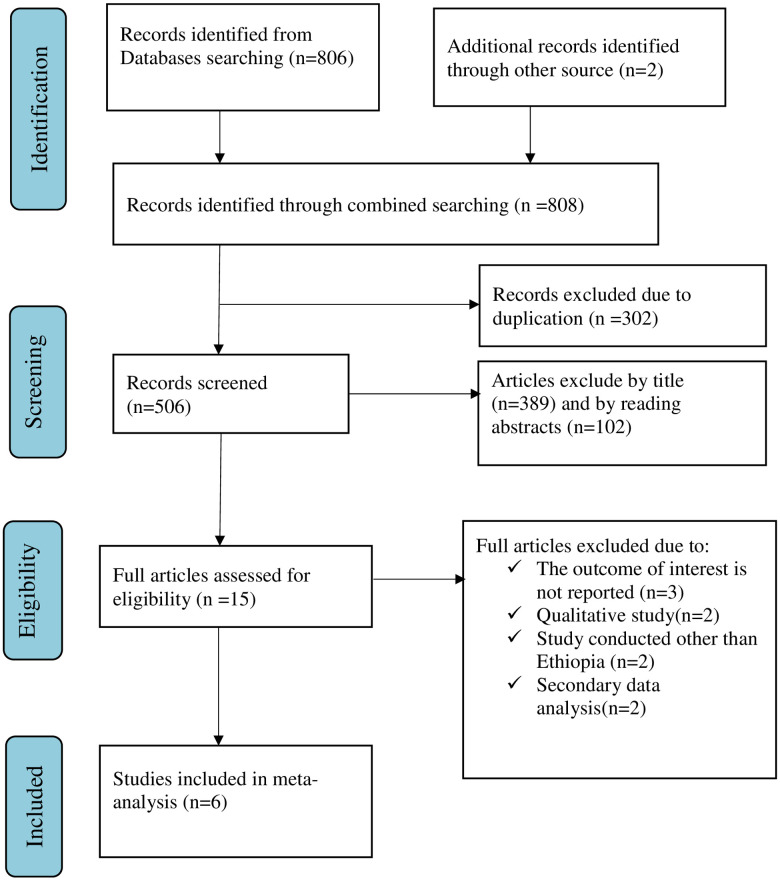
Flow chart of selection for systematic review and meta-analysis on women's awareness of obstetric fistula and its associated factors in Ethiopia, 2022.

The search strategies yielded 808 articles. Later, 302 records were removed due to duplications and (506) articles remained. Articles were excluded by title (*n* = 389) and by reading abstracts (*n* = 102). Thus, 15 full-text papers were accessed and evaluated for inclusion criteria. Nine more articles were excluded, mostly for the reasons in Figure 1. As a result, six studies fulfilled the inclusion criteria to undergo the final systematic review and meta-analysis (Table 1).

**Table 1:** Study characteristics included in the systematic review and meta-analysis on the prevalence of women's awareness of obstetric fistula and its associated factors in Ethiopia.

Authors	Study month	Study year	Year of publication	Region	Sample size	Women's awareness of obstetric fistula (%)	Study design	Study setting	Study quality
Rundasa DN. et al. (29)	December 30–January 31	2021	2021	Oromia	400	50%	CS	Institutional based	7
Asefa Z et al. (30)	February 15–March 15	2020	2020	SNNP	422	40.8%	CS	Community-based cross-sectional study	7
Teklay BA. et al. (31)	March February 26–March 24	2020	2021	Tigray	605	42.15	CS	Community based cross-sectional survey	8
Balcha WF et al. (32)	March 4–29/2019	2019	2020	Amhara	413	39.5	CS	Institutional based	8
Tsega M. et al. (33)	May 1–20	2021	2022	Amhara	784	36.4%	CS	Community-based cross-sectional study	8
Defar S. et al. (34)	March 01–30/2018	2018	2018	Oromia	400	50	CS	Community-based cross-sectional study	7

NB. CS; Cross-sectional, SNNP; Southern Nation Nationalists and People.

### Women's awareness of obstetric fistula in Ethiopia

**Figure 2:**
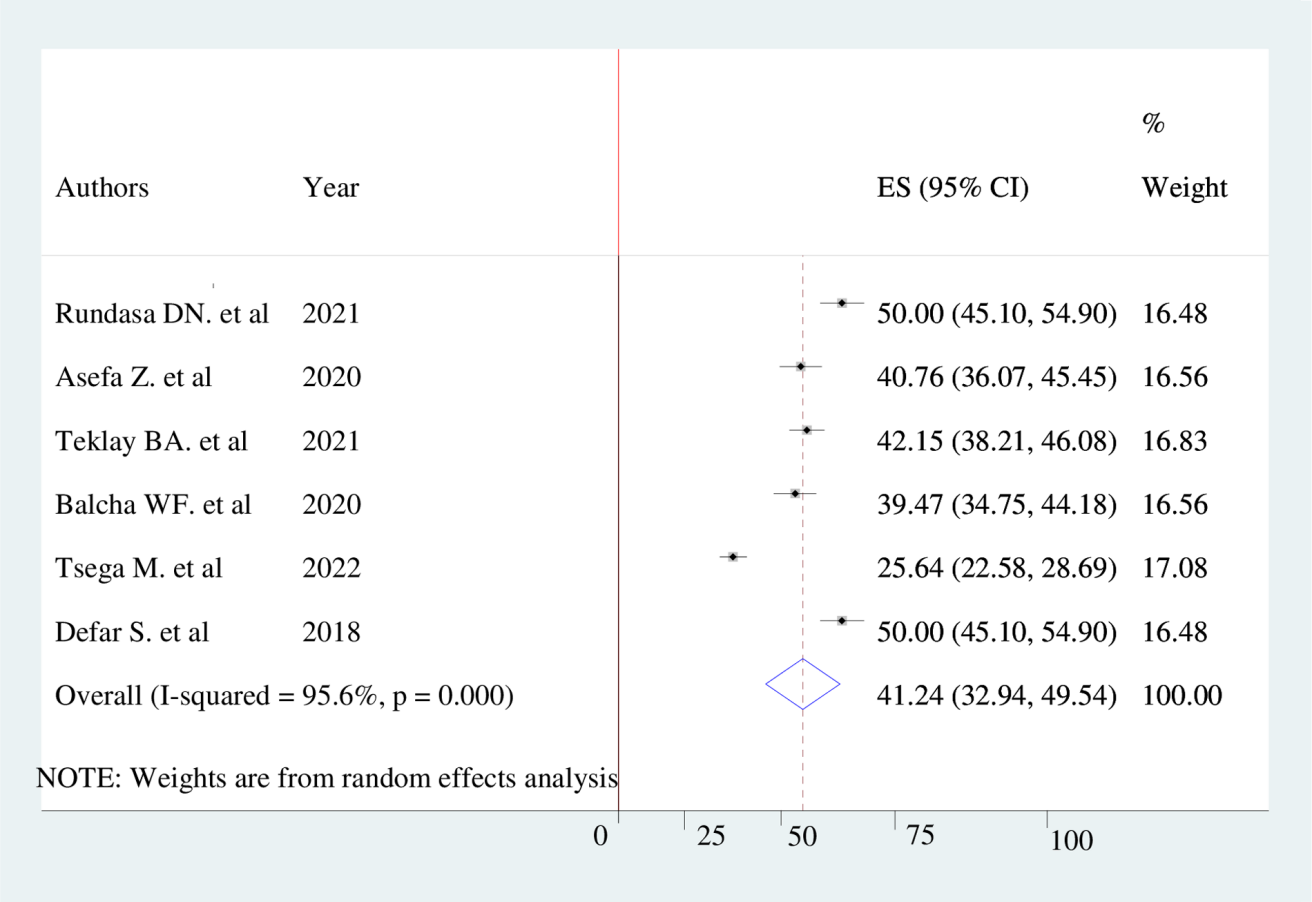
Pooled prevalence of women's awareness of obstetric fistula in Ethiopia, 2022.

The overall level of women's awareness of obstetric fistula in Ethiopia was 41.24% (95% CI: 32.94%−49.54%), with the Cochrane heterogeneity index (*I*^2 ^= 95.6%), *p* = 0.000, showing the substantial objective heterogeneity of different studies (*I*^2 ^> 50%). A forest plot was used to show the overall women's awareness of obstetric fistula and to identify the presence of heterogeneity (Figure 2).

### Publication bias

**Figure 3:**
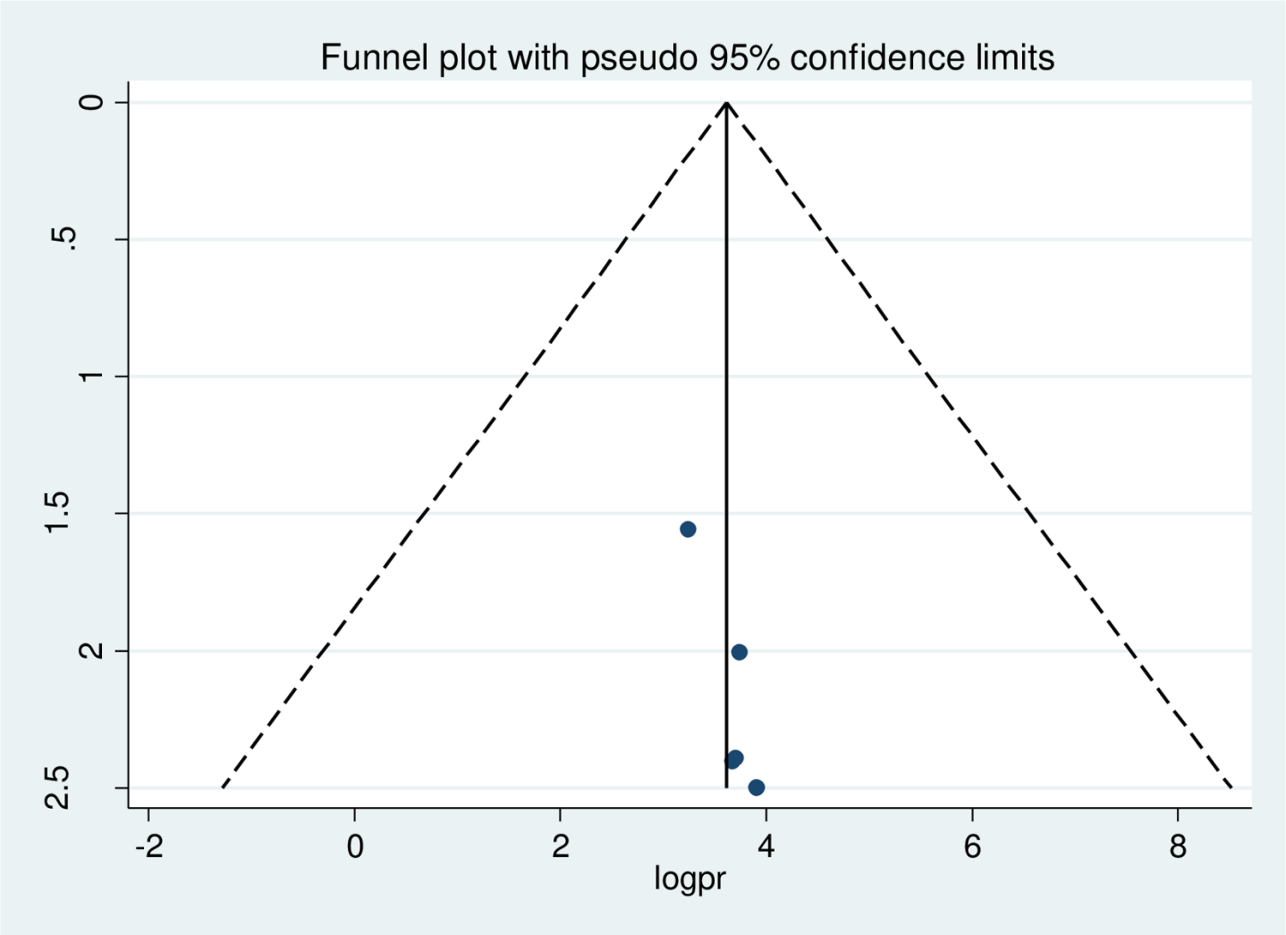
Funnel plot showing the symmetric distribution of articles on women's awareness of obstetric fistula in Ethiopia, 2022.

A funnel plot was used in this systematic review and meta-analysis to test for publication bias at a significance level lower than 0.05. The funnel plot's present no evidence of publication bias which was confirmed by the Egger's regression test, which was not statistically significant (*p* > 0.05) (Figure 3).

### Subgroup analysis of women's awareness of obstetric fistula

**Figure 4:**
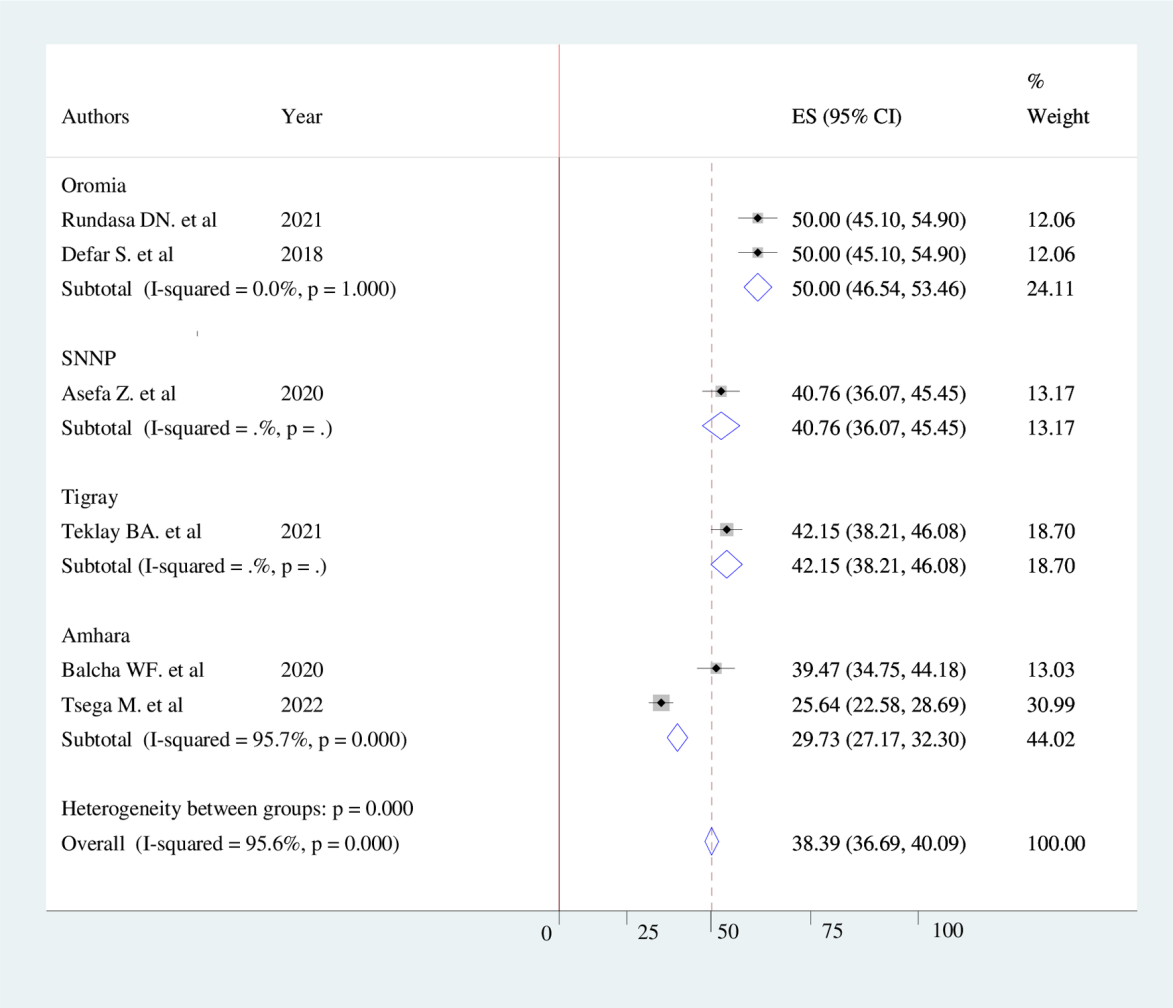
Forest plot showing sub-group analysis of women's awareness of obstetric fistula and its associated factors by region in Ethiopia, 2022.

The result of subgroup analysis by region revealed that the overall level of awareness of women toward obstetric fistula was highest in the Oromia region, at 50% (95% CI: 46.54−53.46) and lowest in the Amhara region, at 29.73% (95% CI: 27.17–32.30) (Figure 4).

### Sensitivity analysis

According to the results of a random effect model, no single study had an impact on the overall level of Ethiopian women's awareness of obstetric fistula (Table 2).

**Table 2:** Sensitivity analysis of studies included in the systematic review and meta-analysis on the prevalence of women's awareness of obstetric fistula in Ethiopia.

Study omitted	Estimate (95% CI)
Rundasa DN et al. (2021)	35.68 (5.81–218.98)
Asefa Z. et al. (2020)	36.64 (5.90–227.52)
Teklay BA. et al. (2021)	36.10 (5.47–238.26)
Balcha WF. et al. (2020)	36.83 (5.94–228.32)
Tsega M. et al. (2022)	43.90 (5.66–340.43)
Defar S. et al. (2018)	35.68 (5.51–218.98)
Combined	37.16 (6.77–203.72)

### Factors associated with women's awareness of obstetric fistula

In the meta-analysis, urban residence, giving birth at a health institution, having secondary and above educational status, having antenatal care follow-up, and participating in pregnant women's conferences were factors associated with women's awareness of obstetric fistula in Ethiopia.

The odds of those residing in urban areas having awareness of obstetric fistula were 2.32 times higher than those residing in rural areas (AOR = 2.32, 95% CI: 1.40–3.85). The odds of those with secondary and above educational status having awareness of obstetric fistula were 3.27 times higher than those with no formal education (AOR = 3.27, 95% CI: 2.15–4.97). The odds of those who give birth at a health institution having awareness of obstetric fistula were 2.84 times higher than those who give birth at home (AOR = 2.84, 95% CI: 1.92–4.21). The odds of those who participate in pregnant women's conferences having awareness of obstetric fistula were 4.64 times higher than their counterparts (AOR = 4.64, 95% CI: 2.88–7.49).

## Discussion

This study shows that the pooled prevalence of women's awareness of obstetric fistula was low. Urban residence, giving birth at a health institution, having secondary and above educational status, having antenatal care follow-up, and participating in pregnant women's conferences were factors associated with women's awareness of obstetric fistula in Ethiopia.

In this study, the level of women's awareness of obstetric fistula (41.24%) is comparable with a study conducted in Northern Ghana (45%) (35). However, this result is much lower than studies conducted in Nigeria (52.0%) and Southern Tanzania (61.1%) (36, 37). Likewise, the pooled level of women's awareness of obstetric fistula is higher than in a national study conducted in Ethiopia (38%) and Burkina Faso (36%) (38, 39). The study time and study population differences could be the cause of the various findings. For instance, national studies in Ethiopia and Burkina Faso were conducted in 2016 and 2013, respectively; since then, many activities have been carried out to raise women's awareness on the issues of obstetric fistula risk factors, prevention, and treatments.

The subgroup analysis between regions indicated that the highest level of good awareness of obstetric fistula was reported in the Oromiya region (50%). The finding is greater than a study done in Kenya (44%) (40). However, it is lower than the study conducted at Mile 4 missionary Hospital, Abakaliki (57.8%) (41). The possible explanation might be due to the study population and study design difference. For instance, a study conducted in Abakaliki was a hospital-based study design. Thus, women who visit health facilities might have a better awareness of maternal and child health services, which might mean they have a better awareness of OBF.

The result of the meta-analysis revealed that the odds of those residing in an urban area having awareness of obstetric fistula were 2.32 times higher than those residing in rural areas. This finding is in line with a study conducted in Burkina Faso (39). A possible explanation might be that women residing in an urban area might have access to quality health facilities and better education, which makes them more likely to have more awareness of obstetric fistula than their counterparts. Therefore, we suggest that particular attention should be paid to women residing in rural areas, particularly focusing on improving women's awareness of the prevention of obstetric fistula, risk factors, and healthcare-seeking behavior.

In this study, women who had completed secondary education and above were 3.27 times more likely to have awareness of obstetric fistula than their counterparts. This result is similar to that attained in a study conducted in Northern Ghana (35). This may be because their education gave them knowledge about their sexual and reproductive health and enabled them to read the methods of preventing obstetric fistula from different websites, magazines, and newspapers.

The odds of those who give birth at a health institution having awareness of obstetric fistula were 2.84 times higher than those who had given birth at home. A possible explanation might be that women who give birth at a health institution are more likely to be exposed to obstetric health care providers and receive counseling on the possible complications of childbirth, including obstetric fistula, compared to women who give birth at home. Thus, we strongly recommend a zero-tolerance approach toward home delivery. Furthermore, stakeholders and health extension workers should promote institutional delivery.

The odds of those who have antenatal care follow-up having awareness of obstetric fistula were 2.73 times higher than those who do not have antenatal care follow-up. A possible explanation might be that women who have antenatal care follow-up might attain knowledge on health promotion and prevention, particularly regarding birth preparedness and complication readiness. In addition, they might be aware of the merits of giving birth at a health institution and have access to essential and emergency obstetric services that prevent the occurrence of prolonged and obstructed labor and the risk of developing obstetric fistula.

The odds of those who participate in pregnant women's conferences having awareness of obstetric fistula were 4.64 times higher than their counterparts. A possible explanation might be that most of the time, pregnant women's conferences focus on complications that arise during pregnancy, childbirth, and the postnatal period. Thus, as obstetric fistula is a complication that arises during childbirth, it might be addressed during pregnant women's conferences, which would facilitate their awareness of obstetric fistula.

## Conclusion

The pooled prevalence of women's awareness of obstetric fistula was low. Over half of the women had poor awareness of obstetric fistula in Ethiopia. Urban residence, giving birth at a health institution, having secondary and above educational status, having antenatal care follow-up, and participating in pregnant women's conferences were the factors associated with women's awareness of obstetric fistula. Therefore, enhancing the awareness of women who reside in rural areas and promoting institutional delivery and antenatal care follow-up is recommended. Furthermore, policymakers and stakeholders should empower women and provide special attention to the neglected but important public health problem that is obstetric fistula.

## Data Availability

The original contributions presented in the study are included in the article/**Supplementary Material**, further inquiries can be directed to the corresponding author.

## Supplementary material

SUPPLEMENTARY FILE S1

Preferred Reporting Items for Systematic Reviews and Meta-Analyses (PRISMA) Guideline.

SUPPLEMENTARY FILE S2

Newcastle-Ottawa Quality Assessment Scale of six cross-sectional studies included in the review of women’s awareness of obstetric fistula and its associated factors in Ethiopia.
