# Supplementary material for: Systematic review and meta-analysis of women's awareness of obstetric fistula and its determinants in Ethiopia
Source: Front Glob Womens Health. 2023 May 19;4:1151083. doi: 10.3389/fgwh.2023.1151083 (PMC10235548; doi:10.3389/fgwh.2023.1151083)
Supplement: Supplementary file 2 [file Table2.docx]

| **Newcastle-Ottawa Quality Assessment Scale scale for cross-sectional studies** | **Selection** | | | | **Comparability** | **Outcome** | | **Total score** |
| --- | --- | --- | --- | --- | --- | --- | --- | --- |
|  | Representativeness (1) | Sample size  (1) | Non-respondents  (1) | Ascertainment of the exposure (risk factor)  (2) | The subjects in different outcome groups are comparable, based on the study design or analysis. confounding factors are controlled (2) | Assessment of the outcome  (2) | Statistical test  (1) |  |
| Rundasa DN. et al 29 | 1 | 1 | 1 | 1 | 1 | 1 | 1 | 7 |
| Asefa Z et al 30 | 1 | 1 | 1 | 1 | 1 | 2 | 1 | 7 |
| Teklay BA. et al 31 | 1 | 1 | 1 | 1 | 1 | 2 | 1 | 8 |
| Balcha WF et al 32 | 1 | 1 | 1 | 2 | 1 | 2 | 1 | 8 |
| Tsega M. et al 33 | 1 | 1 | 1 | 2 | 1 | 2 | 1 | 8 |
| Defar S. et al 34 | 1 | 1 | 1 | 2 | 1 | 2 | 1 | 7 |
| Rundasa DN. et al 29 | 1 | 1 | 1 | 2 | 1 | 2 | 1 | 8 |
